# Pseudomonas Unmasked: Poor Continuous Positive Airway Pressure (CPAP) Hygiene Leads to a Case of Pseudomonas Pneumonia

**DOI:** 10.7759/cureus.77697

**Published:** 2025-01-20

**Authors:** Nur Mando, Daniel Antonious, Nora Gillen, Michelle Joseph, Erica Thomson

**Affiliations:** 1 Internal Medicine, University of Florida College of Medicine/Ascension Sacred Heart, Pensacola, USA; 2 General Surgery, Florida State University College of Medicine, Pensacola, USA

**Keywords:** copd (chronic obstructive pulmonary disease), cpap hygiene, pseudomonas aeroginosa, pseudomonas pneumonia, sleep apnea

## Abstract

Continuous positive airway pressure (CPAP) therapy is commonly prescribed for obstructive sleep apnea. However, improper cleaning and maintenance of CPAP equipment can create an environment that encourages bacterial colonization, leading to opportunistic respiratory infections in patients, especially in immunocompromised patients with concomitant comorbidities such as chronic obstructive pulmonary disease (COPD). We describe the case of a 57-year-old male patient with a history of chronic bronchitis, bronchiectasis, COPD, and obstructive sleep apnea who presented to the emergency department with worsening dyspnea, productive cough, and respiratory distress. Imaging revealed pneumonia and a respiratory pathogen panel identified *Pseudomonas aeruginosa* along with *Moraxella* and *Proteus* species. The patient admitted to poor CPAP maintenance and noted green-colored growth on his mask, consistent with *Pseudomonas* colonization. His symptoms improved with tailored antibiotic therapy. CPAP equipment requires routine cleaning to prevent bacterial growth. *P. aeruginosa*, a waterborne pathogen that thrives in humid environments, can colonize improperly maintained equipment, leading to severe respiratory infections, especially in immunocompromised patients. This case highlights the need for patient education on CPAP hygiene to prevent infectious complications. Further research is needed to clarify the relationship between CPAP use, water quality, and infection risk, thereby informing evidence-based guidelines for both clinicians and patients.

## Introduction

Continuous positive airway pressure (CPAP) is commonly prescribed for the management of obstructive sleep apnea (OSA). CPAP can significantly improve sleep quality and overall health by helping patients maintain a patent airway with consistent airflow during sleep. The mechanism of blowing humidified air into secretory surfaces of the patients, such as the nose and mouth, can potentially lead to infections if the CPAP machine mask is colonized with bacteria. Research shows that colonized nasal spray bottles can lead to respiratory infections [1].

It is essential to properly maintain this equipment, including sanitizing it correctly. Improperly cleaning this equipment leads to the growth of infectious organisms, causing avoidable infectious diseases. Cleaning instructions by the manufacturer in the machine's manual should be reiterated by the healthcare providers prescribing the machine. There may be CPAP users who are not following cleaning instructions or have been improperly educated which leads to improper maintenance allowing for opportunistic organisms to colonize in these humid, damp environments. Research done in the pediatric population shows a discrepancy in patient care of their CPAP machine equipment [2]; however, as an increasing number of adult cases with poor CPAP hygiene-associated pneumonia is identified [3], further research is warranted in this patient population too. The conflicting research on the correlation between serious respiratory illnesses and CPAP only adds to the confusion surrounding patient education. There is no question, however, that improperly cleaning CPAP equipment can lead to respiratory infections [4].

This report describes the case of a patient who acquired pneumonia caused by *Pseudomonas aeruginosa *due to improper CPAP sanitation techniques. We highlight the proper cleaning protocol for CPAP equipment, illustrate the need for further patient education in CPAP machine users, emphasize its importance in clinical practice, and report the conflicting research on this topic.

## Case presentation

A 57-year-old Caucasian male patient with a past medical history of chronic bronchitis, bronchiectasis, chronic obstructive pulmonary disease (COPD), and OSA presented to the Emergency Department (ED) via the Emergency Medical Services (EMS) with dyspnea and cough. He was noted to be tachypneic with a respiratory rate of 40 breaths/minute with 79% oxygen saturation on ambient air. He received breathing treatments with DuoNeb nebulizers along with dexamethasone en route to the hospital. He reported a flu-like illness two weeks prior to arrival, with persistent productive cough, dyspnea, and wheezing along with green sputum production. The patient reported compliance with his home medications, including his home CPAP machine, but had been requiring his as-needed COPD medication more frequently. His dyspnea was intolerable, prompting him to call EMS. 

**Figure 1:**
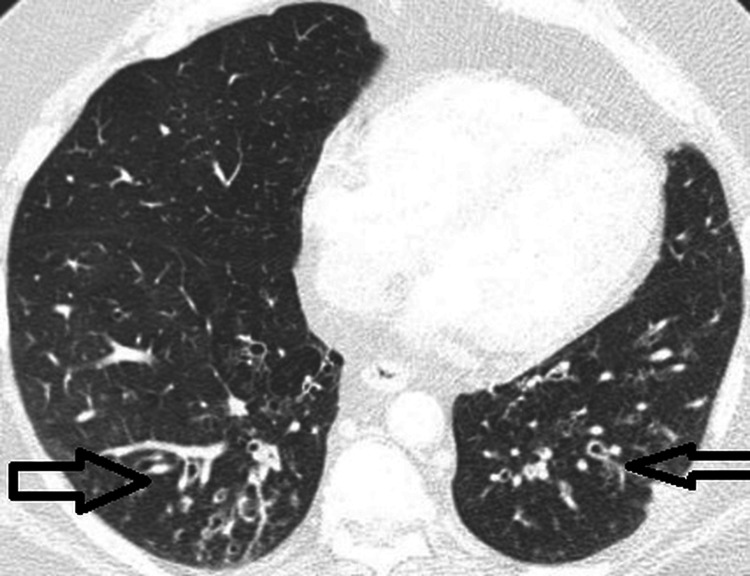
CTA of the chest showing mild patchy bilateral lower lobe pulmonary infiltrate with bronchial wall thickening CTA: computed tomographic angiography

On physical exam, the patient was tachypneic and tachycardic, with diminished breath sounds bilaterally, and was noted to have expiratory wheezes. The patient was started on empiric intravenous (IV) ceftriaxone. A computed tomography angiography (CTA) of the chest was negative for pulmonary embolism but showed mild patchy bilateral lower lobe and right upper lobe pulmonary infiltrates with bronchial wall thickening consistent with pneumonia (Figure 1).

His pneumonia pathogen polymerase chain reaction (PCR) panel was positive for *Pseudomonas*, *Moraxella*, and *Proteus*. Accordingly, the empiric antibiotic regimen was changed to IV cefepime to treat the organisms detected on his pneumonia panel. Upon further questioning, the patient admitted that he did not regularly follow CPAP machine cleaning guidelines, and he had noted a green growth on his CPAP mask most likely consistent with colonies of *Pseudomonas*. Thereafter, he had an uncomplicated hospital course, and he was discharged three days after admission on improvement.

## Discussion

A CPAP machine's accessories consist of a mask, tubing, humidifier, and a filter, all of which attach to the CPAP machine and need regular cleaning. The CPAP unit itself is cleaned on an as-needed basis. The CPAP machine and accessories should be disassembled to clean the various parts properly. The mask should be cleaned daily because it covers the face, mouth, and nose, which provides a humid environment perfect for bacterial growth. A cleaning agent such as mild liquid soap with no fragrances, moisturizers, or antibacterial properties is necessary for appropriate sanitization. Hand washing and air drying are recommended instead of using a dishwasher. The CPAP tubing should be cleaned at least once a week. The humidifier, if there is one, should be deeply cleaned weekly, and the water should be replaced daily. Disposable CPAP filters should be replaced monthly or sooner according to how soiled they become with use. Reusable CPAP filters should be cleaned weekly. It is essential to read the manual of the CPAP machine to understand the recommended cleaning frequency and methods instructed by the manufacturer [5].

There is documented evidence of infectious disease caused by infected CPAP machine equipment. However, very little information regarding the risk of wearing a CPAP machine is known to support decision-making for clinicians and patients. Overall, there is conflicting information about the infectious complications of wearing CPAP machines and whether certain accessories, such as humidifiers, filters, and sterilized water, prevent bacterial infection transmission. A retrospective study explored if there was a longitudinal association between CPAP use and clinically significant respiratory infections and found no association [6]. Another prospective cohort study showed that having a positive culture in the CPAP equipment does not lead to clinical significance of rhinosinusitis in chronic rhinosinusitis patients [7]. The use of filters and humidifiers may decrease the risk of respiratory infections, but there is conflicting evidence that humidifiers aerosolize bacteria, allowing them to colonize. Another study showed that patients with OSA who were being treated with CPAP fitted with humidifiers may be aerosolizing bacteria, putting them at risk for respiratory infections. The study also showed that the use of a hydrophobic filter may prevent the passage of microbes [8]. A retrospective study comparing the rate of upper airway infections (URI) in CPAP users with or without heated humidity showed an increase in URIs compared to those treated conservatively [3]. However, another retrospective, case-controlled study showed no increase in URIs in CPAP machine users and that their choice of machine or mask was noncontributory [9].

A study of the water in convection-type humidifiers demonstrated that no aerosolization occurred and concluded that sterile water is not required in these humidifiers [10]. Properly sterilized water is used to avoid infectious waterborne diseases. The water used in the humidifier of a CPAP machine is changed daily, so it follows that this should be properly sanitized water. Water and humid environments are known to aid in the colonization of *P. aeruginosa *[11], which was the bacteria that caused pneumonia in the current patient. *Pseudomonas* colonies appear green and shiny because the bacteria produce pigments called “pyocyanin”, which are thought to be virulent factors [12]. Thus, they appear green when growing in large quantities on a surface similar to what the patient described as the green film on his mask.

Waterborne illnesses contribute a substantial burden to healthcare costs, so conclusive evidence about the use of sterile water for medical equipment is essential to prevent bacterial infections. Tap water is often used by patients to clean CPAP machines because they are not properly educated. A survey performed in 2021 showed that participants believed that tap water was safe to use in home medical devices [13]. A study on the burden of waterborne diseases and healthcare costs of infectious waterborne diseases in 2021 showed that 7.15 million waterborne illnesses occur annually, costing $3.33 billion dollars in direct healthcare [14]. Most hospitalizations and deaths are caused by bacteria that form biofilms, such as nontuberculous *Mycobacteria*, *Pseudomonas*, and *Legionella*, and cost $2.39 billion annually [14]. Thus, more education and research are required to raise awareness about proper water sanitation techniques for medical equipment.

## Conclusions

The conflicting research associated with CPAP machine use and infectious diseases leads to clinical ambiguity. The research available is unclear with regard to the type of water, the use of a humidifier, and the association between CPAP use and respiratory illnesses. This limits the evidence-based decision-making for clinicians and highlights a gap in CPAP machine use. There is an obvious need for quality research to be completed in this area. There is, however, a clear relationship between poor sanitation of CPAP equipment and respiratory illnesses, so teaching patients how to clean their equipment is essential. The instructions provided by the manufacturer should be followed unless more research is performed and otherwise indicated. Ultimately, this case highlights the importance of clinical providers emphasizing the role of sanitation of CPAP machines to prevent serious infections and enhance long-term health outcomes.
